# Social Family Climate and Family Emotional Communications in the Parental Families of Men with Borderline Personality Disorder (BPD) and Mentally Healthy Men

**DOI:** 10.1192/j.eurpsy.2026.11908

**Published:** 2026-07-31

**Authors:** V. I. Rozhdestvenskiy, V. S. Lupashku

**Affiliations:** Department of Clinical Psychology, Saint Petersburg State Pediatric Medical University, Saint Petersburg, Russian Federation

## Abstract

**Introduction:**

The nature of emotional communications and the social family climate in the parental families of individuals with Borderline Personality Disorder (BPD) are considered a key risk factor for the disorder. It is within the family environment that basic skills of emotion regulation, self-identity, and interpersonal relationships are formed; deficiencies in these areas constitute the core symptoms of BPD.

**Objectives:**

The aim of this study was to identify the relationships between family emotional communications and the social family climate in the parental families of men with BPD compared to control the group of mentally healthy men.

**Methods:**

Data were collected in 2025 using an online form developed by the authors. The sample consisted of 14 men diagnosed with Borderline Personality Disorder **(**BPD) and 20 men in the control group of mentally healthy men, aged 18–25. The Family Emotional Communications Questionnaire (FEC) by Kholmogorova A.B. was used to assess emotional communications, and the Family Environment Scale (FES), adapted for Russian-speaking respondents, was used to evaluate the social family climate.

**Results:**

In the parental families of men with BPD, Family Cohesion was negatively correlated with Criticism (r = -0.81, p < 0.01) and Emotional Invalidation (r = -0.64, p < 0.05). Achievement Orientation was positively correlated with Family Perfectionism (r = 0.8, p < 0.01), Striving for External Well-Being (r = 0.64, p < 0.05), and Over-involvement (r = 0.55, p < 0.05). The Control subscale was positively correlated with Family Perfectionism (r = 0.6, p < 0.05). Over-involvement was positively associated with the Moral-Religious Emphasis (r = 0.69, p < 0.01) and Intellectual-Cultural Orientation (r = 0.56, p < 0.05) subscales. In the control group, Cohesion was negatively correlated with Emotional Invalidation (r = -0.53, p < 0.05). Criticism was negatively correlated with the Conflict subscale (r = -0.56, p < 0.05). Family Perfectionism was positively correlated with Achievement Orientation (r = 0.5, p < 0.05) and negatively with Active-Recreational Orientation (r = -0.48, p < 0.05). Active-Recreational Orientation was negatively correlated with Striving for External Well-Being (r = -0.53, p < 0.05).

**Conclusions:**

The parental families of men with BPD exhibit a specific configuration of the family system, where rigid control, achievement orientation, and emotional coldness form a mutually reinforcing structure that creates a pathogenic developmental environment. In the control group, dysfunctional elements are fragmented and do not form such a cohesive pattern, indicating a fundamentally different quality of the family environment.

**Disclosure of Interest:**

None Declared

